# $f_{(\lambda,\mu)}$-statistical convergence of order *α̃* for double sequences

**DOI:** 10.1186/s13660-017-1512-y

**Published:** 2017-10-04

**Authors:** Mahmut Işik, Yavuz Altin

**Affiliations:** 1 Faculty of Education, Harran University, Şanlıurfa, Turkey; 2 Department of Mathematics, Fırat University, Elazığ, Turkey

**Keywords:** 40A05, 40C05, 46A45, double sequences, statistical convergence, Cesàro summability

## Abstract

New concepts of $f_{\lambda,\mu }$-statistical convergence for double sequences of order *α̃* and strong $f_{\lambda,\mu }$-Cesàro summability for double sequences of order *α̃* are introduced for sequences of (complex or real) numbers. Furthermore, we give the relationship between the spaces $w_{\tilde{\alpha },0}^{2} ( f,\lambda,\mu )$, $w_{\tilde{\alpha }}^{2} ( f,\lambda,\mu ) $ and $w_{\tilde{\alpha},\infty }^{2} ( f,\lambda,\mu )$. Then we express the properties of strong $f_{\lambda,\mu }$-Cesàro summability of order *β̃* which is related to strong $f_{\lambda,\mu }$-Cesàro summability of order *α̃*. Also, some relations between $f_{\lambda,\mu }$-statistical convergence of order *α̃* and strong $f_{\lambda,\mu }$-Cesàro summability of order *α̃* are given.

## Introduction

The first idea of statistical convergence goes back to the first edition of the famous Zygmund’s monograph [1]. The statistical convergence was introduced for real and complex sequences by Steinhaus [2]. Fast [3] extended the usual concept of sequential limit and called it statistical convergence. Schoenberg [4] called it as D-Convergence. The idea depends on a certain density of subsets of $\mathbb{N}$. The natural density (or asymptotic density) of a set *A*⊂ $\mathbb{N}$ is defined by $\delta ( A ) =\lim_{n\rightarrow \infty } \frac{1}{n}\vert \{ k\leq n:k\in A \} \vert $ if the limit exists, where $\vert A(n)\vert $ is cardinality of the set $A(n)$ (see [5]). A sequence *x*= $( x_{k} ) $ of complex numbers is said to be statistically convergent to some number *ℓ* if $\delta ( \{ k\in \mathbb{N}:\vert x_{k}-\ell \vert \geq \varepsilon \} ) $ has natural density zero for $\varepsilon >0$. *ℓ* is necessarily unique, which is statistical limit of $( x_{k} ) $, and written as ${S\mbox{-}\lim x_{k}=\ell }$. The space of all statistically convergent sequences is denoted by *S* (see [5–20]).

The order of statistical convergence of a sequence of positive linear operators was given by Gadjiev and Orhan [21], and after that Çolak [22] introduced statistical convergence of order *α* and strong *p*-Cesàro summability of order *α*.

Statistical convergence was introduced for double sequences by Mursaleen and Edely [23]. Besides this topic was studied by many authors (such as [15, 24, 25]). For some further works in this direction, we refer to [26–30].

The concepts of convergence and statistical convergence for double sequence can be expressed as follows.

Let $s^{2}$ denote the space of all double sequences, and let $\ell_{\infty }^{2}$, $c^{2}$ and $c_{0}^{2}$ be the linear spaces of bounded, convergent and null sequences $x= ( x_{jk} ) $ with complex terms, respectively, normed by $\Vert x\Vert _{ ( \infty,2 ) }=\sup_{j,k}\vert x_{jk}\vert $, where *j*, $k\in \mathbb{N}= \{ 1,2,\, \ldots \} $.

A double sequence $x= ( x_{j,k} ) _{j,k=0}^{\infty }$ has Pringsheim limit *ℓ* provided that for every $\varepsilon >0$ there exists $N\in \mathbb{N} $ such that $\vert x_{j,k}-\ell \vert < \varepsilon $ whenever $j,k>N$. In this case, we write $P\mbox{-}\lim x=\ell $ [31].

$x= ( x_{j,k} ) _{j,k=0}^{\infty }$ is bounded if there exists a positive number *M* such that $\vert x_{j,k}\vert < M$ for all *j* and *k*, that is, $\Vert x\Vert =\sup_{j,k\geq 0}\vert x_{j,k}\vert <\infty $.

Let $K\subseteq \mathbb{N} \times \mathbb{N} $ and $K ( m,n ) = \{ ( j,k ) :j\leq m,k\leq n \} $. The double natural density of *K* is defined by

$$ \delta_{2} ( K ) =P\mbox{-}\lim_{m,n}\frac{1}{mn}\bigl\vert K ( m,n ) \bigr\vert \quad \mbox{if the limit exists.} $$

A double sequence $x= ( x_{jk} ) _{j,k\in \mathbb{N}}$ is said to be statistically convergent to *ℓ* if for every $\varepsilon >0$ the set $\{ ( j,k ) :j\leq m,k\leq n:\vert x_{jk}- \ell \vert \geq \varepsilon \} $ has double natural density zero [23]. In this case, one can write $st_{2}\mbox{-}\lim x=\ell $, and we denote the collection of all statistically convergent double sequences by $st_{2}$. Recently, Çolak and Altin [27] introduced double statistically convergent of order *α*, and they examined some inclusion relations.

The idea of a modulus function was introduced in 1953 by Nakano [32]. Later, Ruckle [33] and Maddox [34] used this concept to construct some sequence spaces. Let us remind modulus function.

*f* $: [ 0,\infty ) \rightarrow [ 0,\infty ) $ is called a modulus function if

1. $f ( x ) =0$ if and only if $x=0$,
2. $f ( x+y ) \leq f ( x)+f(y ) $ for every $x,y\in \mathbb{R}^{+}$,
3. *f* is increasing,
4. *f* is continuous from the right at 0.

Hence, *f* must be continuous everywhere on $[ 0,\infty ) $. A modulus function may be bounded or unbounded. For example, $f ( x ) =\frac{x}{1+x}$ is bounded, but $f ( x ) =x ^{p}$, $0< p\leq 1$ is unbounded.

Aizpuru *et al.* [35] introduced and discussed the concepts of *f*-statistical convergence and *f*-statistically Cauchy sequences, a single sequence of numbers, where *f* is an unbounded modulus function. Bhardwaj and Dhawan [36] continued this work and defined *f*-statistical convergence of order *α*. This new idea was introduced by Borgohain and Savaş [37] under the name of ’$f_{\lambda }$-statistical convergence’. Aizpuru *et al.* also studied these concepts for double sequences [38]. Mursaleen [39] introduced *λ*-statistical convergence as an extension of $( V, \lambda ) $-summability of Leindler [40] with the help of a non-decreasing sequence, $\lambda = ( \lambda_{n} ) $ being a non-decreasing sequence of positive numbers tending to ∞ with $\lambda_{n+1}\leq \lambda_{n}+1$, $\lambda _{1}=1$. The generalized de la Vallee-Poussin mean is defined by

$$ t_{n} ( x ) =\frac{1}{\lambda_{n}}{\sum_{k\in I_{n}}} x_{k}, $$

where $I_{n}= [ n-\lambda_{n}+1,n ] $.

*λ*-statistical convergence of double sequences has been expressed by Mursaleen *et al.* [41].

## $f_{\lambda,\mu }$-double statistical convergence of order *α̃*

In this section, we introduce $f_{\lambda,\mu }$-double statistical convergence of order *α̃* for double sequences.

Throughout this paper, we take $s,t,u,v\in ( 0,1 ] $ as otherwise indicated. We will write *α̃* instead of $( s,t ) $ and *β̃* instead of $( u,v ) $. Also, we define the following:

$$\begin{aligned}& \widetilde{\alpha } \preceq \widetilde{\beta }\quad \Longleftrightarrow \quad s \leq u\quad \mbox{and}\quad t\leq v, \\& \widetilde{\alpha } \prec \widetilde{\beta }\quad \Longleftrightarrow \quad s< u\quad \mbox{and}\quad t< v, \qquad \qquad \qquad \qquad \qquad \qquad \qquad \qquad \qquad \qquad \\& \widetilde{\alpha } \cong \widetilde{\beta }\quad \Longleftrightarrow \quad s=u\quad \mbox{and}\quad t=v, \\& \widetilde{\alpha } \in ( 0,1 ] \quad \Longleftrightarrow \quad s, t \in ( 0,1 ], \\& \widetilde{\beta } \in ( 0,1 ] \quad \Longleftrightarrow \quad u, v \in ( 0,1 ], \\& \widetilde{\alpha } \cong 1\quad \mbox{in case }s=t=1, \\& \widetilde{\beta } \cong 1\quad \mbox{in case }u=v=1, \\& \widetilde{\alpha } \succ 1\quad \mbox{in case }s>1\quad \mbox{and}\quad t>1. \end{aligned}$$

Furthermore, we write $S_{\tilde{\alpha }}^{2} ( f,\lambda, \mu ) $ to denote $S_{ ( s,t ) }^{2} ( f,\lambda,\mu ) $ and $S_{\widetilde{\beta }}^{2}( f,\lambda,\mu ) $ to denote $S_{ ( u,v ) }^{2} ( f,\lambda, \mu ) $ in the section below.

We begin with the following definitions.

Let $\lambda = ( \lambda_{n} ) $ and $\mu = ( \mu_{m} ) $ be two non-decreasing sequences of positive real numbers tending to ∞ with $\lambda_{n+1}\leq \lambda_{n}+1$, $\lambda_{1}=0; \mu_{n+1}\leq \mu_{n}+1$, $\mu_{1}=0$ and $\widetilde{\alpha }\in ( 0,1 ] $ be given.

Let $K\subseteq \mathbb{N}\times \mathbb{N}$ *be* a two-dimensional set of positive integers and *f* be an unbounded modulus function. Then $\delta_{\tilde{\alpha }}^{{f}2} ( {\lambda,\mu } ) $-double $density$ of *K* is defined as

$$ \delta_{\tilde{\alpha }}^{{f}2}(K)=\lim_{n,m\rightarrow \infty } \frac{1}{f ( \lambda_{n}^{s}\mu_{m}^{t} ) }f \bigl( \bigl\vert \bigl\{ (j,k) \in I_{n}\times I_{m}: ( i,j ) \in K\bigr\} \bigr\vert \bigr) \quad \mbox{if the limit exists.} $$

### Definition 2.1

Let $\lambda = ( \lambda_{n} ) $ and $\mu = ( \mu_{m} ) $ be two non-decreasing sequences of positive real numbers as above and $\widetilde{\alpha }\in ( 0,1 ] $ be given.

$( x_{jk} ) $ is said to be $f_{\lambda,\mu }$-statistically convergent of order *α̃* if there is a complex number *ℓ* such that, for every $\varepsilon >0$,

$$ \lim_{n,m\rightarrow \infty }\frac{1}{f ( \lambda_{n}^{s}\mu_{m} ^{t} ) }f \bigl( \bigl\vert \bigl\{ (j,k) \in I_{n}\times I_{m}: \vert x_{jk}-\ell \vert \geq \varepsilon \bigr\} \bigr\vert \bigr) =0. $$

In this case we write $S_{\tilde{\alpha }}^{2} ( f,\lambda, \mu ) \mbox{-}\lim_{j,k}x_{jk}=\ell $, and we denote the set of all $f_{\lambda,\mu }$-statistically convergent double sequences of order *α̃* by $S_{\tilde{\alpha }}^{2} ( f, \lambda,\mu ) $, where *f* is an unbounded modulus function.

In the case of $f(x)=x$, $\widetilde{\alpha }\cong 1$ and $\lambda _{n}=n$, $\mu_{m}=m$, $f_{\lambda,\mu }$-statistical convergence of order *α̃* reduces to the statistical convergence of double sequences [23]. If $x= ( x_{jk} ) $ is $f_{\lambda,\mu }$-statistically convergent of order *α̃* to the number *ℓ*, then *ℓ* is determined uniquely. $f_{\lambda,\mu }$-double statistical convergence of order *α̃* is well defined for $\widetilde{\alpha }\in ( 0,1 ] $ but it is not well defined for $\widetilde{\alpha }\succ 1$. For this, let us define $x= ( x_{jk} ) $ as follows:

$$ x_{jk}= \textstyle\begin{cases} 1,&\mbox{if }j+k \mbox{ even}, \\ 0,&\mbox{if }j+k\mbox{ odd}. \end{cases} $$

Since $\lim_{t\rightarrow \infty }\frac{f ( t ) }{t}>0$, we have

$$\begin{aligned} & \lim_{n,m\rightarrow \infty }\frac{1}{f ( \lambda_{n}^{s}\mu _{m}^{t} ) }f \bigl( \bigl\vert \bigl\{ (j,k)\in I_{n}\times I_{m}:\vert x_{jk}-1\vert \geq \varepsilon \bigr\} \bigr\vert \bigr) \leq \lim_{n,m\rightarrow \infty }\frac{f ( [ \vert \lambda _{n}^{s}\mu_{m}^{t}\vert ] ) +1}{f ( 2\lambda _{n}^{s}\mu_{m}^{t} ) }=0 \end{aligned}$$

and

$$\begin{aligned} & \lim_{n,m\rightarrow \infty }\frac{1}{f ( \lambda_{n}^{s}\mu _{m}^{t} ) }\bigl\vert \bigl\{ (j,k)\in I_{n}\times I_{m} : \vert x_{jk}-0 \vert \geq \varepsilon \bigr\} \bigr\vert \leq \lim_{n,m\rightarrow \infty }\frac{f ( [ \vert \lambda _{n}^{s}\mu_{m}^{t}\vert ] ) +1}{f ( 2\lambda _{n}^{s}\mu_{m}^{t} ) }=0 \end{aligned}$$

for $\widetilde{\alpha }\succ 1$, that is, $s>1$ and $t>1$, so that $x= ( x_{jk} ) $ is $f_{\lambda,\mu }$-statistically convergent of order *α̃* both to 1 and 0, i.e., $S_{\tilde{\alpha }}^{2} ( f,\lambda,\mu ) \mbox{-}\lim x_{jk}=1$ and $S_{\tilde{\alpha }}^{2} ( f,\lambda,\mu ) \mbox{-}\lim x_{jk}=0$. But this is impossible.

### Theorem 2.2

*Let* *f* *be an unbounded modulus function and* $\widetilde{\alpha }\in ( 0,1 ] $. *Let* $x= ( x_{jk} ) $, $y= ( y_{jk} ) $ *be any two sequences of complex numbers*. *Then*

(i) *If* $S_{\tilde{\alpha }}^{2} ( f,\lambda,\mu ) \mbox{-}\lim x _{jk}=\ell_{0}$ *and* $c\in \mathbb{C}$, *then* $S_{\tilde{\alpha }}^{2} ( f,\lambda,\mu ) \mbox{-}\lim cx_{jk}=c\ell_{0}$;
(ii) *If* $S_{\tilde{\alpha }}^{2} ( f,\lambda,\mu ) \mbox{-} \lim x_{jk}=\ell_{o}$ *and* $S_{\tilde{\alpha }}^{2} ( f,\lambda, \mu ) \mbox{-}\lim y_{jk}=\ell_{1}$, *then* $S_{\tilde{\alpha }}^{2} ( f,\lambda,\mu ) \mbox{-}\lim ( x_{jk}+y_{jk} ) =\ell _{0}+\ell_{1}$.

### Theorem 2.3

*Let* *f* *be an unbounded modulus function and* $\tilde{\alpha },\tilde{\beta }$ *be two real numbers such that* 0⪯ $\widetilde{\alpha }\preceq \widetilde{\beta }\preceq 1$. *Then* $S_{\tilde{\alpha }}^{2} ( f,\lambda,\mu ) \subseteq S_{\tilde{\beta }}^{2} ( f,\lambda,\mu ) $ *and strict inclusion may occur*.

### Proof

Let $\tilde{\alpha },\tilde{\beta }\in (0,1]$ be given such that $\tilde{\alpha }\leq \tilde{\beta }$. Since *f* is increasing, we have

$$\begin{aligned} &\frac{1}{f ( \lambda_{n}^{u}\mu_{m}^{v} ) } f \bigl( \bigl\vert \bigl\{ (j,k) \in I_{n} \times I_{m}:\vert x_{jk}-\ell \vert \geq \varepsilon \bigr\} \bigr\vert \bigr) \\ & \quad \leq \frac{1}{f ( \lambda_{n}^{s}\mu_{m}^{t} ) }f \bigl( \bigl\vert \bigl\{ (j,k)\in I_{n} \times I_{m}:\vert x_{jk}- \ell \vert \geq \varepsilon \bigr\} \bigr\vert \bigr) \end{aligned}$$

for every $\varepsilon >0$, and this gives $S_{\tilde{\alpha }}^{2} ( f,\lambda,\mu ) \subseteq S_{\tilde{\beta }}^{2} ( f, \lambda,\mu ) $. To show that the strict inclusion may occur, consider a sequence $x= ( x_{jk} ) $ defined by

$$ x_{jk=} \textstyle\begin{cases} jk, & \mbox{if }n- [ \vert \lambda_{n}\vert ] +1 \leq j\leq n\mbox{ and }m- [ \vert \mu_{m}\vert ] +1\leq k\leq m, \\ 0, & \mbox{otherwise} \end{cases} $$

and we take $f ( x ) =x^{p}$, $( 0< p\leq 1 ) $ and hence $x\in S_{\tilde{\beta }}^{2} ( f,\lambda,\mu ) $ for $\tilde{\beta }\in (\frac{1}{2},1]$, (i.e., $\frac{1}{2}< u\leq 1$ and $\frac{1}{2}< v\leq 1$ ), but $x\notin S_{\tilde{\alpha }}^{2} ( f, \lambda,\mu ) $ for *α̃* $\in (0,\frac{1}{2}]$ (i.e., $0< s\leq \frac{1}{2}$ and $0< t\leq \frac{1}{2}$ ). □

The following results can be easily derived from Theorem 2.3.

### Corollary 2.4

*If* $x= ( x_{jk} ) $ *is* $f_{\lambda, \mu }$-*statistically convergent of order* *α̃* *to* *ℓ*, *for some* *α̃* *such that* $\widetilde{\alpha }\in ( 0,1 ] $, *then it is* $f_{\lambda, \mu }$-*statistically convergent to* *ℓ*, *and the inclusion is strict*.

### Corollary 2.5

*Let* $\widetilde{\alpha },\widetilde{\beta } \in ( 0,1 ] $ *be given*. *Then*

(i) $S_{\widetilde{\alpha }}^{2} ( f,\lambda,\mu ) =S_{ \widetilde{\beta }}^{2} ( f,\lambda,\mu ) $ *if* $\widetilde{\alpha }\cong \widetilde{\beta }$.
(ii) $S_{\widetilde{\alpha }}^{2} ( f,\lambda,\mu ) =S ^{2} ( f,\lambda,\mu ) $ *if* $\widetilde{\alpha }\cong 1$.

## Strongly double Cesàro summability of order *α̃* defined by a modulus function

In this section, we give the relationships between the spaces $w_{\tilde{\alpha },0}^{2} ( f,\lambda,\mu ) ,w_{\tilde{\alpha }}^{2} ( f,\lambda,\mu ) $ and $w_{ \tilde{\alpha },\infty }^{2} ( f,\lambda,\mu ) $.

### Definition 3.1

Let *f* be a modulus function and *α̃* be a positive real number. We have

$$\begin{aligned}& w_{\tilde{\alpha },0}^{2} ( f,\lambda,\mu ) = \biggl\{ x=(x _{jk})\in s^{2}:\lim_{n,m\rightarrow \infty } \frac{1}{ ( \lambda _{n}\mu_{m} ) ^{\tilde{\alpha }}}{\sum_{j\in J_{n}}} {\sum _{k\in I_{n}}} f \bigl( \vert x_{jk}\vert \bigr) =0 \biggr\} , \\& w_{\tilde{\alpha }}^{2} ( f,\lambda,\mu ) = \biggl\{ x=(x _{jk})\in s^{2}:\lim_{n,m\rightarrow \infty } \frac{1}{ ( \lambda _{n}\mu_{m} ) ^{\tilde{\alpha }}}{ \sum_{j\in J_{n}}} { \sum _{k\in I_{n}}} f \bigl( \vert x_{jk}-\ell \vert \bigr) =0 \biggr\} , \\& w_{\tilde{\alpha },\infty }^{2} ( f,\lambda,\mu ) = \biggl\{ x=(x_{jk})\in s^{2}:\sup_{n,m} \frac{1}{ ( \lambda_{n}\mu _{m} ) ^{\tilde{\alpha }}}{ \sum_{j\in J_{n}}} {\sum _{k\in I_{n}}} f \bigl( \vert x_{jk}\vert \bigr) < \infty \biggr\} . \end{aligned}$$

### Theorem 3.2

(i) *Let* *f* *be a modulus function*. *For* $\tilde{\alpha }\succ 0$, *we have* $w_{\tilde{\alpha },0}^{2} ( f, \lambda,\mu ) \subset $ $w_{\tilde{\alpha },\infty }^{2} ( f,\lambda,\mu ) $.
(ii) *Let* *f* *be a modulus function*. *For* *α̃* ⪰1, *we have* $w_{\tilde{\alpha }}^{2} ( f, \lambda,\mu ) \subset $ $w_{\tilde{\alpha },\infty }^{2} ( f,\lambda,\mu ) $.

### Proof

(i) The proof of (i) is trivial.

(ii) Let $x\in w_{\tilde{\alpha }}^{2} ( f,\lambda, \mu ) $. By the definition of modulus function (ii) and (iii), we have

$$\begin{aligned}& \frac{1}{ ( \lambda_{n}\mu_{m} ) ^{\tilde{\alpha }}}{ \sum_{j\in J_{n}}} {\sum _{k\in I_{n}}} f \bigl( \vert x_{jk}\vert \bigr) \leq \frac{1}{ ( \lambda_{n}\mu_{m} ) ^{\tilde{\alpha }}} {\sum_{j\in J_{n}}} {\sum _{k\in I_{n}}} f \bigl( \vert x_{jk}-\ell \vert \bigr) +f \bigl( \vert \ell \vert \bigr) \frac{1}{ ( \lambda_{n}\mu _{m} ) ^{\tilde{\alpha }}}{ \sum _{j\in J_{n}}} {\sum_{k\in I_{n}}} 1, \end{aligned}$$

and since *α̃* ⪰1 and $x\in w_{\tilde{\alpha }} ^{2} ( f,\lambda,\mu ) $, we have $x\in w_{ \tilde{\alpha },\infty }^{2} ( f,\lambda,\mu ) $, which completes the proof. □

### Theorem 3.3

*For any modulus function* *f* *and* *α̃* ⪰1, *we have* $w_{\tilde{\alpha }}^{2} ( \lambda,\mu ) \subset $ $w_{\tilde{\alpha }}^{2} ( f, \lambda,\mu ) $, $w_{\tilde{\alpha },0}^{2} ( \lambda, \mu ) \subset $ $w_{\tilde{\alpha },0}^{2} ( f,\lambda, \mu ) $ *and* $w_{\tilde{\alpha },\infty }^{2} ( \lambda, \mu ) \subset $ $w_{\tilde{\alpha },\infty }^{2} ( f,\lambda,\mu ) $.

### Proof

We give the proof only when $w_{\tilde{\alpha }, \infty }^{2} ( \lambda,\mu ) \subset $ $w_{\tilde{\alpha }, \infty }^{2} ( f,\lambda,\mu ) $ and the rest of cases will follow similarly. Let $x\in w_{\tilde{\alpha },\infty }^{2} ( \lambda,\mu ) $, so that

$$ \sup_{n,m} \frac{1}{ ( \lambda_{n}\mu_{m} ) ^{\tilde{\alpha }}}{ \sum _{j\in J_{n}}} {\sum_{k\in I_{n}}} \vert x_{jk}\vert < \infty. $$

Let $\varepsilon >0$ and choose *δ* with $0<\delta <1$ such that $f ( t ) <\varepsilon $ for $0\leq t<\delta $. Now we write

$$ \frac{1}{ ( \lambda_{n}\mu_{m} ) ^{\tilde{\alpha }}}{ \sum_{j\in J_{n}}} { \sum _{k\in I_{n}}} f \bigl( \vert x_{jk}\vert \bigr) =\sum _{1}+\sum_{2}, $$

where the first summation is over $\vert x_{jk}\vert \leq \delta $ and the second is over $\vert x_{jk}\vert > \delta $. Then $\sum_{1}\leq \varepsilon\cdot\frac{1}{ ( \lambda_{n} \mu_{m} ) ^{\tilde{\alpha }-1}}$ and, for $\vert x_{jk}\vert > \delta $, we use the fact that

$$ \vert x_{jk}\vert < \frac{\vert x_{jk}\vert }{ \delta }< 1+ \biggl[ \biggl\vert \frac{\vert x_{jk}\vert }{ \delta }\biggr\vert \biggr], $$

where $[ \vert t\vert ] $ denotes the integer part of *t*. Given $\varepsilon >0$, by the definition of *f*, we have

$$ f \bigl( \vert x_{jk}\vert \bigr) \leq \biggl( 1+ \biggl[ \biggl\vert \frac{\vert x_{jk}\vert }{\delta }\biggr\vert \biggr] \biggr) f ( 1 ) \leq 2f ( 1 ) \frac{\vert x _{jk}\vert }{\delta } $$

for $\vert x_{jk}\vert >\delta $ and hence $\sum_{2} \leq 2f ( 1 ) \delta^{-1}{ \sum_{j\in J_{n}}} { \sum_{k\in I_{n}}} \vert x_{jk}\vert $, which together with $\sum_{1}\leq \varepsilon \frac{1}{ ( \lambda_{n}\mu _{m} ) ^{\tilde{\alpha }-1}}$ yields

$$ \frac{1}{ ( \lambda_{n}\mu_{m} ) ^{\tilde{\alpha }}}{ \sum_{j\in J_{n}}} { \sum _{k\in I_{n}}} f \bigl( \vert x_{jk}\vert \bigr) \leq \varepsilon \cdot\frac{1}{ ( \lambda_{n}\mu_{m} ) ^{ \tilde{\alpha }-1}}+2f ( 1 ) \delta^{-1}\frac{1}{ ( \lambda_{n}\mu_{m} ) ^{\tilde{\alpha }}}{ \sum_{j\in J_{n}}} { \sum_{k\in I_{n}}} \vert x_{jk}\vert . $$

Since $\tilde{\alpha }\geq 1$ and $x\in w_{\tilde{\alpha },\infty } ^{2} ( \lambda,\mu ) $, we have $x\in w_{\tilde{\alpha }, \infty }^{2} ( f,\lambda,\mu ) $ and the proof is complete. □

### Theorem 3.4

*Let* *f* *be a modulus function* *f* *and* $\tilde{\alpha }\succ 0$. *If* $\lim_{t\rightarrow \infty }\frac{f ( t ) }{t}>0$, *then* $w_{\tilde{\alpha }}^{2} ( f, \lambda,\mu ) \subset w_{\tilde{\alpha }}^{2}( \lambda,\mu )$.

### Proof

Following the proof of Proposition 1 of Maddox [42], we have $l=\lim_{t\rightarrow \infty }\frac{f ( t ) }{t}= \inf \{ \frac{f ( t ) }{t}:t>0 \} $. By the definition of *l*, we have $f ( t ) \geq lt$ for all $t\geq 0$. Since $l>0$, we get $t\leq l^{-1}f ( t ) $ for all $t\geq 0$, and so

$$ \frac{1}{ ( \lambda_{n}\mu_{m} ) ^{\tilde{\alpha }}}{ \sum_{j\in J_{n}}} { \sum _{k\in I_{n}}} \vert x_{jk}-\ell \vert \leq l ^{-1}\frac{1}{ ( \lambda_{n}\mu_{m} ) ^{\tilde{\alpha }}} { \sum_{j\in J_{n}}} { \sum_{k\in I_{n}}} f \bigl( \vert x_{jk}-\ell \vert \bigr), $$

from where it follows that $x\in w_{\tilde{\alpha }}^{2} ( f, \lambda,\mu ) $ whenever $x\in w_{\tilde{\alpha }}^{2} ( \lambda,\mu ) $. □

### Theorem 3.5

*For any modulus* *f* *such that* $\lim_{t\rightarrow \infty }\frac{f ( t ) }{t}>0$ *and* *α̃* ⪰1. *Then* $w_{\tilde{\alpha }}^{2} ( \lambda,\mu ) =w_{\tilde{\alpha }}^{2} ( f,\lambda,\mu ) $.

## Relation between $f_{\lambda,\mu }$-statistical convergence of order *α̃* and strongly double Cesàro summability of order *α̃* defined by a modulus function

In this section, we give the relationship between the strong $f_{\lambda,\mu }$-Cesàro summability of order *α̃* and $f_{\lambda,\mu }$-statistical convergence of order *β̃*.

### Lemma 4.1

*Let* *f* *be an unbounded function such that there is a positive constant* *c* *such that* $f ( xy ) \geq cf ( x ) f ( y ) $ *for all* $x\geq 0$, $y\geq 0$ [42].

### Theorem 4.2

*Let* $0\prec \tilde{\alpha }\preceq \tilde{\beta }\preceq 1$ *and* *f* *be an unbounded modulus function such that there is a positive constant* *c* *such that* $f ( xy ) \geq cf ( x ) f ( y ) $ *for all* $x\geq 0$, $y\geq 0$ *and* $\lim_{t\rightarrow \infty }\frac{f ( t ) }{t}>0$. *If a sequence* $x=(x_{jk})$ *is strongly* $f_{\lambda,\mu }$-*Cesàro summable of order* *α̃* *with respect to* *f* *to* *ℓ*, *then it is* $f_{\lambda,\mu }$-*statistically convergent of order* *β̃* *to* *ℓ*.

### Proof

For any sequence $x=(x_{jk})$ and $\varepsilon >0$, using the definition of modulus function (ii) and (iii), we have

$$\begin{aligned} { \sum_{j\in J_{n}}} { \sum_{k\in I_{n}}} f \bigl( \vert x_{jk}-L\vert \bigr) & \geq f \biggl( { \sum _{j\in J_{n}}} { \sum_{k\in I_{n}}} \vert x_{jk}-\ell \vert \biggr)\geq f \bigl( \bigl\vert \bigl\{ (j,k)\in I_{n}\times I_{m} : \vert x_{jk}-\ell \vert \geq \varepsilon \bigr\} \bigr\vert \varepsilon \bigr) \\ & \geq cf \bigl( \bigl\vert \bigl\{ (j,k)\in I_{n}\times I_{m}:\vert x _{jk}-\ell \vert \geq \varepsilon \bigr\} \bigr\vert f( \varepsilon ) \bigr) \end{aligned}$$

and since *α̃* ⪯ *β̃*

$$\begin{aligned} & \frac{1}{n^{s}m^{t}}{ \sum_{j=1}^{n}} { \sum_{k=1}^{m}} f \bigl( \vert x_{jk}-\ell \vert \bigr) \\ & \quad \geq \frac{1}{n^{s}m^{t}}cf \bigl( \bigl\vert \bigl\{ (j,k)\in I_{n} \times I_{m} : \vert x_{jk}-\ell \vert \geq \varepsilon \bigr\} \bigr\vert f ( \varepsilon ) \bigr) \\ &\quad \geq \frac{1}{n^{u}m^{v}}cf \bigl( \bigl\vert \bigl\{ (j,k)\in I_{n} \times I_{m} :\vert x_{jk}-\ell \vert \geq \varepsilon \bigr\} \bigr\vert f ( \varepsilon ) \bigr) \\ & \quad =\frac{1}{n^{u}m^{v}f ( n^{u}m^{v} ) }cf \bigl( \bigl\vert \bigl\{ (j,k) \in I_{n}\times I_{m} :\vert x_{jk}-\ell \vert \geq \varepsilon\bigr\} \bigr\vert f ( \varepsilon ) \bigr) f \bigl( n^{u}m^{v} \bigr), \end{aligned}$$

where, using the fact that $\lim_{t\rightarrow \infty }\frac{f ( t ) }{t}>0$ and $x\in w_{\tilde{\alpha }}^{2} ( f, \lambda,\mu ) $, it follows that $x\in S_{\tilde{\beta }}^{2} ( \lambda,\mu ) $ and the proof is complete. □

If we take *β̃* ≅*α̃* in Theorem 4.2, we have the following.

### Corollary 4.3

*Let* *f* *be an unbounded modulus function* $f ( xy ) \geq cf ( x ) f ( y ) $, *where* *c* *is a positive constant for all* $x\geq 0$, $y\geq 0$ *and* $\lim_{t\rightarrow \infty }\frac{f ( t ) }{t}>0$ *and* $\tilde{\alpha }\in (0,1]$. *If a sequence is strongly* $f_{\lambda, \mu }$-*Cesàro summable of order* *α̃* *with respect to* *f* *to* *ℓ*, *then it is* $f_{\lambda,\mu }$-*statistically convergent of order* *α̃* *to* *ℓ*.

## Conclusions

In this study, we define $f_{\lambda,\mu }$-statistical convergence for double sequences of order *α̃*, where *f* is an unbounded modulus function. Besides this we also study strong $f_{\lambda,\mu }$-Cesàro summability for double sequences of order *α̃* and give inclusion relations. These results are the generalizations of the studies by Meenakshi *et al.* [43].
